# A High-Definition View of Functional Genetic Variation from Natural Yeast Genomes

**DOI:** 10.1093/molbev/msu037

**Published:** 2014-01-14

**Authors:** Anders Bergström, Jared T. Simpson, Francisco Salinas, Benjamin Barré, Leopold Parts, Amin Zia, Alex N. Nguyen Ba, Alan M. Moses, Edward J. Louis, Ville Mustonen, Jonas Warringer, Richard Durbin, Gianni Liti

**Affiliations:** ^1^Institute for Research on Cancer and Ageing, Nice (IRCAN), University of Nice, Nice, France; ^2^The Wellcome Trust Sanger Institute, Cambridge, United Kingdom; ^3^Donnelly Centre for Cellular and Biomolecular Research, University of Toronto, Toronto, ON, Canada; ^4^Department of Cell & Systems Biology, University of Toronto, Toronto, ON, Canada; ^5^Stanford Center for Genomics and Personalized Medicine, Stanford University School of Medicine; ^6^Centre of Genetic Architecture of Complex Traits, University of Leicester, Leicester, United Kingdom; ^7^Department of Chemistry and Molecular Biology, University of Gothenburg, Gothenburg, Sweden

**Keywords:** population genomics, functional variation, genome evolution, yeast, subtelomeres, loss-of-function variants

## Abstract

The question of how genetic variation in a population influences phenotypic variation and evolution is of major importance in modern biology. Yet much is still unknown about the relative functional importance of different forms of genome variation and how they are shaped by evolutionary processes. Here we address these questions by population level sequencing of 42 strains from the budding yeast *Saccharomyces cerevisiae* and its closest relative *S. paradoxus*. We find that genome content variation, in the form of presence or absence as well as copy number of genetic material, is higher within *S. cerevisiae* than within *S. paradoxus*, despite genetic distances as measured in single-nucleotide polymorphisms being vastly smaller within the former species. This genome content variation, as well as loss-of-function variation in the form of premature stop codons and frameshifting indels, is heavily enriched in the subtelomeres, strongly reinforcing the relevance of these regions to functional evolution. Genes affected by these likely functional forms of variation are enriched for functions mediating interaction with the external environment (sugar transport and metabolism, flocculation, metal transport, and metabolism). Our results and analyses provide a comprehensive view of genomic diversity in budding yeast and expose surprising and pronounced differences between the variation within *S. cerevisiae* and that within *S. paradoxus*. We also believe that the sequence data and de novo assemblies will constitute a useful resource for further evolutionary and population genomics studies.

## Introduction

A central issue in biology is how genetic variation influences variation in organismal phenotypes, as well as how this variation is shaped by evolutionary processes. So far, the emphasis in population genomics studies has been on single-nucleotide polymorphisms (SNPs), which are the most abundant form of sequence variation and therefore the most informative about population history. However, increasing attention is being devoted to other forms of variation including structural, genome content, and copy number variation (CNV), which might be more likely to have large phenotypic effects (Conrad et al. 2010; Sudmant et al. 2010). Little is known, however, about the relative biological importance of these various forms of variation in different species. The Baker’s yeast *Saccharomyces cerevisiae* has emerged as an attractive system in which to address these questions, having long served as an important model organism for molecular biology and genetics but also for comparative genomics and the study of genome evolution (Cliften et al. 2003; Kellis et al. 2003; Dujon et al. 2004; Dujon 2010; Hittinger 2013). Recent years have seen a growing interest in using *S. cerevisiae* and its closest relatives to study natural variation, ecology, and population level genetics and genomics (Replansky et al. 2008; Liti and Schacherer 2011; Hittinger 2013) as well to exploit natural variation in the study of the genetic architecture of traits (Liti and Louis 2012). In one of the first population genomics studies, we revealed the strong population structures of *S. cerevisiae* and its closest relative *S. paradoxus*, with most variants being private to specific phylogenetic lineages, and demonstrated the influence of human activity and domestication on the evolution of the former but not the latter species (Liti, Carter, et al. 2009). A number of other studies have corroborated and extended these findings in various directions (Doniger et al. 2008; Schacherer et al. 2009; Zhang et al. 2010; Hyma et al. 2011; Warringer et al. 2011; Dunn et al. 2012; Hyma and Fay 2013), such that a coherent framework for understanding the population structure and evolutionary history of Baker’s yeast is now emerging.

A question of primary interest in yeast population genomics has been the extent to which the highly stratified genetic structure is driven by on the one hand geography and on the other hand by ecology and adaptation to different lifestyles and environmental niches. So far, the evidence points in favor of geography being the most important factor (Liti, Carter, et al. 2009; Cromie et al. 2013; Hittinger 2013). *Saccharomyces paradoxus* populations found in different parts of the world are highly diverged at the sequence level, do not seem to have recently exchanged genetic material, and in some cases display partial reproductive isolation (Sniegowski et al. 2002; Koufopanou et al. 2006; Liti, Carter, et al. 2009). The genetic structure of *S. cerevisiae* is similarly characterized by strong geographical stratification but is nuanced by a larger degree of admixture between populations and the presence of strains with phylogenetically mosaic genomes (Liti, Carter, et al. 2009; Cromie et al. 2013). This admixture has likely been facilitated by the association of *S. cerevisiae* with human fermentation practices and deliberate or accidental dispersal of domesticated strains to different parts of the world (Hyma and Fay 2013). Overall, genetic divergence is much higher in *S. paradoxus* than in *S. cerevisiae*, the most highly diverged strains in the former species being separated by ∼3.5% at the sequence level as compared to 0.5–0.8% in the latter (Liti, Carter, et al. 2009). Surprisingly, however, phenotypic diversity is substantially higher in *S. cerevisiae* than in *S. paradoxus* (Liti, Carter, et al. 2009; Warringer et al. 2011). This observation is highly unexpected under a model where phenotypic variation is primarily the product of gradual accumulation of SNPs throughout the genome. Therefore, it raises the hypothesis that other evolutionary processes, potentially involving other forms of genetic variation, are responsible.

Several advances have been made in the search for the genetic variants that underlie phenotypic variation in yeast, primarily in *S. cerevisiae*. Due to the high degree of population stratification, genome-wide association studies are problematic to carry out in natural yeast populations (Connelly and Akey 2012), but studies utilizing recombinant populations obtained by crossing diverged parental strains have proven fruitful (Liti and Louis 2012). Quantitative trait loci (QTLs) have been identified for a range of traits including the ability to grow at high temperature (Steinmetz et al. 2002; Sinha et al. 2008; Parts et al. 2011), resistance to numerous chemical compounds (Ehrenreich et al. 2010, 2012), and enological traits (Marullo et al. 2007; Ambroset et al. 2011; Salinas et al. 2012). Although most genotype–phenotype mapping has been performed using the same small set of parent strains with a focus on laboratory strains, an increasing number of studies are utilizing additional strains that cover more of the genetic diversity within the species (Wenger et al. 2010; Cubillos et al. 2011; Parts et al. 2011; Ehrenreich et al. 2012). Less attention has been devoted to the genetic architecture of traits in *S. paradoxus*, though QTLs for telomere length have been identified (Liti, Haricharan, et al. 2009). Little is known about the evolutionary forces that shape the relationship between genotype and phenotype in natural yeast populations. It has been proposed that the predominance of asexual growth and self-fertilization in the natural life history of yeast (Ruderfer et al. 2006; Tsai et al. 2008) makes genetic drift the dominant force driving phenotypic evolution, such that deleterious variants become fixed in specific lineages following repeated population bottlenecks (Warringer et al. 2011; Zörgö et al. 2012). However, there are also studies reporting some evidence for a role of positive selection (Fraser et al. 2010).

So far, population genomics analyses in yeast have mostly relied on microarray technology (Schacherer et al. 2007, 2009) and low-coverage capillary sequencing (Liti, Carter, et al. 2009). A number of individual strain genomes have also been sequenced for various purposes, mostly using capillary and pyrosequencing (Wei et al. 2007; Borneman et al. 2008; Doniger et al. 2008; Argueso et al. 2009; Novo et al. 2009; Dowell et al. 2010; Akao et al. 2011; Borneman et al. 2011; Nijkamp et al. 2012; Ralser et al. 2012; Zheng et al. 2012). Due to rapid technological advances, next-generation sequencing technologies generating short reads have become the most cost-effective choice for population-level whole-genome sequencing. Here we apply high-coverage Illumina sequencing to 42 natural strains from *S. cerevisiae* and *S. paradoxus*. In both species, strains were selected from the set of strains previously surveyed using low-coverage capillary sequencing (Liti, Carter, et al. 2009) and comprehensive phenotypic analyses (Warringer et al. 2011) to be as informative as possible about overall genetic diversity. We construct de novo assemblies and perform analyses to advance our understanding of genomic diversity and evolution, functional variation, and the genotype–phenotype relationship. In addition to SNPs, this data enable analyses of other kinds of variants, such that we can begin to address directly the aforementioned questions about the nature and evolution of genomic variation and its potential phenotypic relevance. We furthermore hope that the sequence data and assemblies will constitute a useful addition to the growing set of resources available for population genomics and genotype–phenotype studies in yeast. Data and results from the project are accessible at http://www.moseslab.csb.utoronto.ca/sgrp/ (last accessed January 14, 2014). 

## Results and Discussion

### De Novo Yeast Genome Assemblies from Short-Read Sequencing Data

We selected 42 haploid (or homozygous diploid) yeast strains and sequenced their genomes to intermediate-to-high (10–60×) coverage using paired-end Illumina technology. A subset of six strains were sequenced to very high (400–800×) coverage (table 1). Genomes of 14 *S. cerevisiae* and 13 *S. paradoxus* strains for which the coverage was higher than 20× after quality filtering were de novo assembled. The six strains with very high coverage were used to explore the effect of sequencing coverage on de novo assembly quality by assembling data subsets systematically downsampled to different coverage levels. We find that assembly quality increases with increasing coverage up to but not above a level of about 120× (supplementary fig. S1, Supplementary Material online). As almost all strains were previously sequenced to low coverage (1–4×) using Sanger technology (Liti, Carter, et al. 2009) and paired-end libraries with long inserts (mean insert size 4,480 bp), we could improve our assemblies by including this data. Mapping Sanger reads onto the Illumina assemblies and performing scaffolding based on paired-end information improved assembly connectivity substantially with an increase in average scaffold N50 from 64.8 to 118.1 kb (table 1).

**Table 1.** Sequencing and De Novo Assembly of Yeast Strain Genomes.

Strain	Subpopulation	Source	Location	Cov^a^	Number of Scaffolds^b^	Assembly Size^b^	Contig N50^b^	Max. Scaffold Size^b^	Scaffold N50^b^
*S. cerevisiae*									
UWOPS87-2421	Mosaic	*Opuntia* spp*.*	Hawaii	821	559/536	11,658,429/11,671,772	103,653/108,473	546,126/546,126	160,939/200,122
UWOPS83-787.3	Mosaic	*Opuntia* spp*.*	Bahamas	627	513/495	11,676,943/11,678,122	113,702/113,702	557,771/885,033	187,881/244,661
YPS128	North American	*Quercus alba*	USA	64	855/791	11,741,720/11,768,379	94,895/99,341	451,294/518,960	109,555/187,623
SK1	Mosaic	Soil	USA	56	1,315/1,119	11,704,669/11,753,398	49,149/54,072	321,072/569,610	67,234/267,272
L1528	Wine/European	Wine	Chile	49	951/808	11,565,290/11,594,025	36,108/39,574	246,181/463,986	55,077/101,460
W303	Mosaic	Laboratory	USA	48	1,391/1,219	11,630,510/11,666,829	34,537/37,986	209,223/284,547	52,228/122,824
DBVPG6765	Wine/European	Unknown	Unknown	46	967/779	11,622,418/11,670,953	46,113/51,348	280,644/540,506	65,667/208,326
Y12	Sake	Sake	Japan	44	1,721/1,582	11,610,064/11,649,747	25,846/26,998	113,242/229,213	36,944/60,148
DBVPG1106	Wine/European	Grapes	Australia	43	1,280/1,163	11,539,734/11,580,105	26,613/27,246	172,964/183,080	36,291/47,371
Y55	Mosaic	Grapes	France	38	1,516/1,219	11,635,423/11,701,139	24,282/25,808	263,008/418,695	42,157/141,898
DBVPG6044	West African	Bili wine	West Africa	35	1,137/983	11,598,603/11,656,308	42,143/45,282	236,917/350,970	52,144/94,331
YJM975	Wine/European	Clinical	Italy	33	3,069/2,429	11,406,731/11,688,902	5,701/5,849	33,464/59,403	7,414/15,038
UWOPS03-461.4	Malaysian	Bertram palm	Malaysia	32	3,646/3,213	11,499,146/11,694,268	5,691/5,844	40,466/60,006	6,919/10,747
DBVPG1373	Wine/European	Soil	Netherlands	32	1,232/970	11,559,454/11,626,504	21,249/22,464	185,406/368,851	35,542/98,526
YJM978	Wine/European	Clinical	Italy	32	—	—	—	—	—
DBVPG1788	Wine/European	Soil	Finland	30	—	—	—	—	—
L1374	Wine/European	Wine	Chile	26	—	—	—	—	—
BC187	Wine/European	Wine	USA	23	—	—	—	—	—
YJM981	Wine/European	Clinical	Italy	16	—	—	—	—	—
*S. paradoxus*									
Y8.5	European	*Quercus* spp*.*	UK	502	439/	11,623,026/	111,332/	547,620/	204,111/
Z1.1	European	*Quercus* spp*.*	UK	423	426/412	11,616,631/11,624,232	113,350/115,435	555,399/555,399	238,384/289,391
Y9.6	European	*Quercus* spp*.*	UK	408	743/	11,615,713/	67,180/	271,892/	83,447/
Z1	European	*Quercus* spp*.*	UK	367	685/	11,686,590/	102,641/	357,366/	128,755/
Q59.1	European	*Quercus* spp*.*	UK	54	931/803	11,714,439/11,757,637	67,680/73,926	362,301/496,642	71,043/150,995
N-44	Far Eastern	*Quercus* spp*.*	Russia	51	2,058/1488	11,576,182/11,733,376	11,054/12,242	66,254/156,128	13,806/36,398
YPS138	American	*Q. velutina*	USA	43	2,047/1529	11,613,204/11,740,006	11,025/11,801	69,807/117,759	14,797/33,285
S36.7	European	*Quercus* spp*.*	UK	42	1,153/1112	11,658,851/11,672,986	46,580/46,880	174,638/187,287	507,27/56,021
Y6.5	European	*Quercus* spp*.*	UK	41	1,134/981	11,655,419/11,704,717	47,030/52,107	197,044/357,414	50,244/78,826
Y7	European	*Quercus* spp*.*	UK	39	1,164/976	11,653,944/11,721,355	43,703/46,679	163,882/230,460	45,872/88,762
Q95.3	European	*Quercus* spp*.*	UK	35	1,229/1,002	11,660,442/11,734,036	42,716/46,351	190,059/307,994	48,261/111,311
UFRJ50816	American	*Drosophila* spp*.*	Brazil	34	—	—	—	—	—
T21.4	European	*Quercus* spp*.*	UK	34	1,187/991	11,656,698/11,699,313	35,788/41,410	131,328/340,019	40,273/70,866
IFO1804	Far Eastern	*Quercus* spp*.*	Russia	26	—	—	—	—	—
W7	European	*Quercus* spp*.*	UK	23	2,070/	11,647,694/	12,927/	94,615/	14,511/
Q74.4	European	*Quercus* spp*.*	UK	23	—	—	—	—	—
Q89.8	European	*Quercus* spp*.*	UK	22	—	—	—	—	—
Q62.5	European	*Quercus* spp*.*	UK	20	—	—	—	—	—
Q69.8	European	*Quercus* spp*.*	UK	18	—	—	—	—	—
Y8.1	European	*Quercus* spp*.*	UK	16	—	—	—	—	—
KPN3829	European	*Quercus* spp*.*	Russia	16	—	—	—	—	—
Q32.3	European	*Quercus* spp*.*	UK	12	—	—	—	—	—

Note.—Strains without values were not de novo assembled. Additional information of the sequenced strains was reported in Liti, Carter, et al. (2009). The *S. cerevisiae* Y12 originally reported from Ivory Coast Palm wine subsequently clarified (Fay J, personal communication) that the strain sent was the Sake strain K12 from Japan.

^a^Refers to raw coverage.

^b^Values before and after forward slashes correspond to before and after additional scaffolding with low coverage paired-end Sanger data. All values in units of base-pairs.

The 27 de novo assemblies have total sizes between 11.58 and 11.77 Mb, suggesting little variability in yeast genome size. This is 3.2–4.8% smaller than the completed *S. cerevisiae* reference genome of 12.16 Mb, indicating a slight underestimation of true genome sizes, presumably due to collapse in the assemblies of transposable elements and perhaps a few other repeat regions. Underestimates are compatible with a reported Ty transposon content of 1–3% in these strains and a higher transposon content of 3.5% in the reference strain S288c (Liti, Carter, et al. 2009). For the majority of strains, the largest scaffolds are longer than the smallest chromosomes of the reference genome (table 1). Some scaffolds in the higher coverage assemblies correspond to near full-length chromosomes. This illustrates that high-quality assembly of yeast genomes is achievable using short-read sequencing data. The structurally complex subtelomeric regions did not generally assemble well however (with some exceptions; see below), a known problem in genome assembly.

### Assembly Scaffolding by Genetic Linkage Reveals Extensive Structural Conservation except in Subtelomeres

Four of the *S. cerevisiae* strains sequenced, the North American YPS128, the West African DBVPG6044, the Sake/Japanese Y12 and the Wine/European DBVPG6765, have been used as parents for advanced intercross lines from which large numbers of highly recombined offspring genomes have been sequenced (Cubillos et al. 2013; Illingworth et al. 2013). The genetic linkage that manifests between nearby loci in these artificial populations was here put to use to further scaffold the de novo assemblies of these four strains. This resulted in chromosome sized scaffolds for all 16 nuclear chromosomes in these strains, containing 94.6–96.1% of the total assembly sequence, and scaffold N50 values of 852–890 kb.

**Fig. 1.**
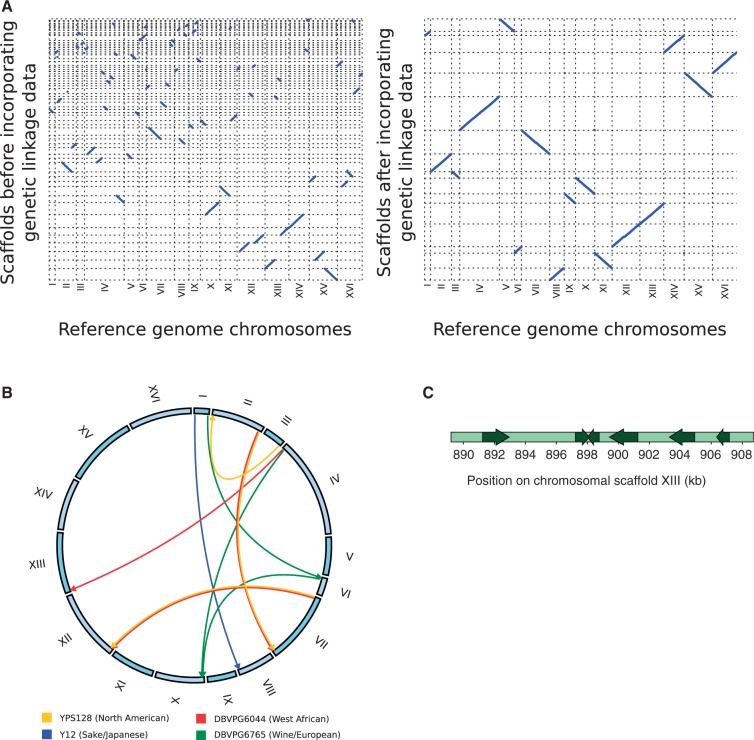
Yeast genome structures revealed by de novo assemblies augmented by genetic linkage data. (*A*) Scaffolding de novo assemblies using genetic linkage information from advanced intercross lines dramatically improves assembly connectivity and reveals extensive structural conservation of the core chromosomes in four of the major *S. cerevisiae* lineages. Displayed is a dot plot of sequence similarity between the assembly scaffolds of the strain YPS128 from the North American phylogenetic lineage and the 16 nuclear chromosomes of the *S. cerevisiae* reference genome (strain S288c), before and after the incorporation of the genetic linkage data into the scaffolding process. After scaffolding by genetic linkage, the majority of the assembly sequence is contained in 16 large scaffolds that are collinear with the chromosomes of the reference genome. Results are highly similar for the other three strains for which genetic linkage data is available; the West African strain DBVPG6044, the Wine/European strain DBVPG6765 and the sake/Japanese strain Y12 (the recent sequencing of the sake strain Kyokai no. 7 (Akao et al. 2011) revealed two intrachromosomal inversions in chromosomes V and XIV in relation to the reference strain S288c, however these are not shared by the sake strain Y12 sequenced here). Only scaffolds bigger than 50 kb are displayed. (*B*) Structural rearrangements relative to the chromosome organization of the *S. cerevisiae* reference genome, all localized to the subtelomeric regions. A directed arrow indicates that a sequence region is aligning to the part of the reference genome where the arrow starts but in the de novo assembly is located in the part of the genome corresponding to where the arrow ends. (*C*) A subtelomeric 18-kb region that assembled well in several strains and could be localized by genetic linkage is displayed with coordinates corresponding to the YPS128 chromosome XIII scaffold. Six genes were found in this region by ab initio gene prediction (arrows indicate coding direction).

The linkage-assisted assemblies exposed the near-complete colinearity and structural conservation of the genomes of four of the major phylogenetic lineages in *S. cerevisiae* (fig. 1*A*). This is consistent with high rates of meiotic spore viability observed in crosses between these strains (Cubillos et al. 2011), as large-scale structural variation would impair proper segregation of chromosomes, as well as the high degree of karyotype conservation within the *S. sensu stricto* species clade (Fischer et al. 2000; Liti et al. 2013). Interestingly, the high degree of structural conservation does not extend into subtelomeric regions, consistent with their rapid evolution and high variability (Liti et al. 2005; Brown et al. 2010). Lower assembly quality in these regions makes analysis difficult; however, the genetic linkage data allowed the identification of some cases of structural variation in the subtelomeres relative to the *S. cerevisiae* reference genome (fig. 1*B*). We also find subtelomeric material that is present in some strains and absent in others, for instance, a segment of approximately 18 kb that localizes to the right subtelomere of chromosome XIII and assembled well in the North American and West African *S. cerevisiae* strains (fig. 1*C*). This genomic region is absent from the Wine/European, Sake/Japanese as well as the *S. cerevisiae* reference strain while being present in all *S. paradoxus* strains, constituting an example of a subtelomeric region that has recently been lost in certain *S. cerevisiae* strains. These kind of structural differences have implications for QTL mapping studies that rely on a reference genome, because the causative sequence responsible for a phenotypic association might in fact be located in a different subtelomere in the mapping strains or simply be absent from the reference genome, thus, risking that the search for candidates is directed to the incorrect genomic region (Cubillos et al. 2011).

### Genome and Gene Content Variation in *S. cerevisiae* Exceeds That in *S. paradoxus*

**Fig. 2.**
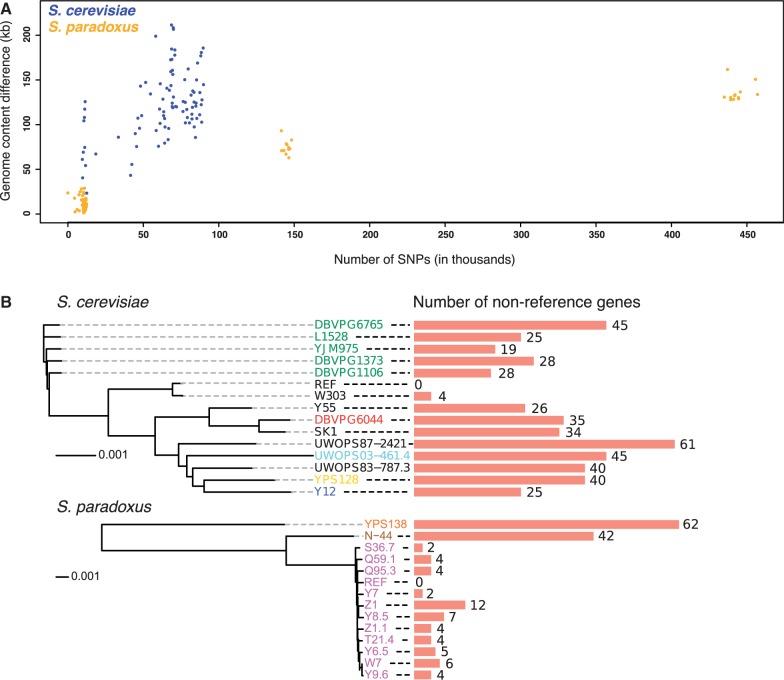
Genome content variation within natural yeast populations. (*A*) The relationship between genetic distance between strains as measured in SNPs and the amount of genomic material being present/absent between strains. All pairwise strain comparisons within each of the two species are included. (*B*) The number of nonreference genes found in each strain genome. Strain colors denote subpopulation origin (for *S. cerevisiae*: green = Wine/European, red = West African, cyan = Malaysian, yellow = North American, dark blue = Sake/Japanese, black = mosaic genome; for *S. paradoxus*: orange = American, brown = Far Eastern, magenta = European). The strain trees are neighbor-joining trees based on genome-wide SNP distances and the scale bars indicate sequence distance in units of SNPs per basepair (distance scales differ between the species).

High quality de novo genome assemblies enable the systematic identification of long sequence segments that are present in only a subset of yeast strains, such as the region exemplified in figure 1*C*. We made pairwise comparisons between strain genomes and summed the total length of sequence regions larger than 1 kb that are present in one strain but not the other and refer to this as genome content variation. In *S. cerevisiae* this sum is always larger than the number of SNPs between a pair of strains for all possible strain comparisons. This is not the case for *S. paradoxus*, surprisingly, as the amount of genome content variation within this species is lower than within *S. cerevisiae*, despite genetic variation in the form of SNPs being almost an order of magnitude larger (fig. 2*A*). In both species there is a positive correlation between the SNP distance between strains and the amount of genome content difference, but the correlation is much weaker in *S. cerevisiae* than in *S. paradoxus* (*r* = 0.51 and *r* = 0.97, respectively). The highly variable relationship in *S. cerevisiae* suggests that this kind of variation is the product of a different mode of evolution than the clocklike nature of SNP accumulation. Nonetheless, clustering strains based on genome content differences recapitulates the known population structures of both species (supplementary fig. S2, Supplementary Material online). To ensure that the detected differences reflects true underlying genome variation, we validated the presence and absence of 14 variable regions across strains by polymerase chain reaction (PCR), confirming the de novo assembly predictions in 326/327 cases, as well as compared one of our assemblies to an alternative assembly for the same strain and found no differences (see supplementary fig. S3, Supplementary Material online and Materials and Methods). The observation that genome content variation is relatively larger within *S. cerevisiae* than within *S. paradoxus* is highly unexpected under a neutral model of genome evolution and implies pronounced differences in the evolutionary histories of these two species. Although challenging to establish at present, we suggest that this unexpected excess of genome content variation in *S. cerevisiae* is likely to be a major contributor to the equally surprising excess of phenotypic variability within this species. We furthermore note that there are considerable amounts of genomic sequence that is present in one or more natural strains but absent in the *S. cerevisiae* reference strain S288c (supplementary fig. S4, Supplementary Material online).

We annotated the de novo assemblies by homology and synteny comparisons to the reference genome. Out of 5,774 nondubious open reading frames (ORFs) in the S288c *S. cerevisiae* reference genome, we recovered a median of 5,417 ORFs (94%) per strain, with most failures to recover a gene appearing to be caused by collapse in the assemblies of very close paralogs. We also identified genes that are not present in the reference genome. The number of nonreference genes present in a *S. cerevisiae* strain genome ranges from four in the lab strain W303, which is very closely related to the reference strain S288c, to 61 in the mosaic strain UWOPS87-2421, with a median of 31 genes per strain (fig. 2*B*). Using the four *S. cerevisiae* strains for which genetic linkage data allowed assembly of chromosome-sized scaffolds, we estimate that at least 75% of these nonreference genes are located in the subtelomeric parts of the chromosomes. By exploiting distant sequence similarity to functionally annotated reference genome proteins, we find that the set of nonreference genes is enriched for gene ontology terms related to flocculation and sugar—particularly maltose—transport and metabolism. These results are consistent with findings on the evolutionary properties of subtelomeric genes across larger evolutionary distances (Brown et al. 2010).

### CNV Is Extensive in Subtelomeres and Greater in *S. cerevisiae* than in *S. paradoxus*

Whereas the analysis described in the previous section is only powered to detect binary presence and absence due to the tendency of similar copies to collapse during de novo assembly, by mapping reads to the reference genome we can also assay differences between higher copy numbers. We identified CNV across all strains with a mapped coverage of 8× or higher (18 *S. cerevisiae* strains and 19 *S. paradoxus* strains). The total size of genomic regions exhibiting CNV between strains is three times larger in *S. cerevisiae* than in *S. paradoxus* (423 kb vs. 142 kb, corresponding to 3.5% and 1.2% of the total genome size, respectively), despite lower levels of overall genetic divergence in the former species. Although the true amount of CNV in *S. paradoxus* could be slightly underestimated due to the quality of the reference genome being lower than that of *S. cerevisiae* (containing multiple gaps where CNV detection is not possible), this is unlikely to explain the large difference observed (see Materials and Methods). These results mirror the recent finding of differences in CNV rates between the great ape lineages (Sudmant et al. 2013). CNV similarity between strains strongly correlates with SNP similarity within both *S. cerevisiae* and *S. paradoxus* (*r* = 0.843 and *r* = 0.885, respectively), and clustering strains based on CNV profiles recapitulates the broad phylogenetic structures of the two species (supplementary fig. S5, Supplementary Material online). In both *S. cerevisiae* and *S. paradoxus* we find very limited CNV in nonsubtelomeric regions and extensive variation in the subtelomeric regions. In *S. cerevisiae*, 32.0% of subtelomeric nucleotide positions are affected by CNV compared with 0.7% in nonsubtelomeric regions (42-fold enrichment), and in *S. paradoxus* the corresponding numbers are 9.3% and 0.04%, respectively (23-fold enrichment). In *S. cerevisiae,* genes contained in regions displaying CNV are enriched for gene ontology terms related to sugar transport and metabolism, flocculation and ion and metal transport and metabolism. The same trends of enrichment are observed in *S. paradoxus* (although with fewer categories reaching statistical significance as a consequence of the overall lower number of genes affected). Other genes with variable copy number between strains in both species include the high copy number subtelomeric *YRF1* helicase, *PAU* seripauperin, and *DUP* gene families. These results imply that largely similar evolutionary forces are shaping the landscapes of CNV in these two species. By considering aggregate sequencing depth across close paralogs in the reference genome, we could unveil further trends not necessarily apparent when considering genes one by one. This gene family based analysis showed that one clinically isolated strain from the Wine/European phylogenetic cluster, YJM981, harbors an extremely large number of *YRF1* gene copies; on the order of 1,000 copies, compared with estimates of 10–40 copies in other strains including the reference genome. This radical copy number increase is consistent with an experimentally demonstrated phenomenon where the subtelomeric Y′-element, which contains the *YRF1* genes, is amplified to serve as an alternative mechanism for telomere maintenance following failure of the conventional telomerase system (Lundblad and Blackburn 1993). This has dramatic consequences on subtelomeric structure and overall genome size, and we confirmed by pulsed field gel electrophoresis that the chromosomes of YJM981 are much larger than normal (supplementary fig. S6, Supplementary Material online). Another closely related clinical isolate, YJM978, also harbors an usually large number of *YRF1* copies, though much fewer than YJM981 (∼150 copies). While we have not identified any obvious loss-of-function mutations affecting the key telomere maintenance genes in these strains, some of them (*EST1*, *EST2*, *RIF2*, *TEL1*, *yKu80*) appear to have very low expression levels in an RNA-seq data set (Skelly et al. 2013). These results raise the intriguing possibility that this alternative mode of telomere elongation actually occurs in natural strains, which to our knowledge has never been demonstrated.

### Convergent Evolution of Subtelomeric CNV Underlies Natural Arsenic Resistance in *S. cerevisiae* and *S. paradoxus*

**Fig. 3.**
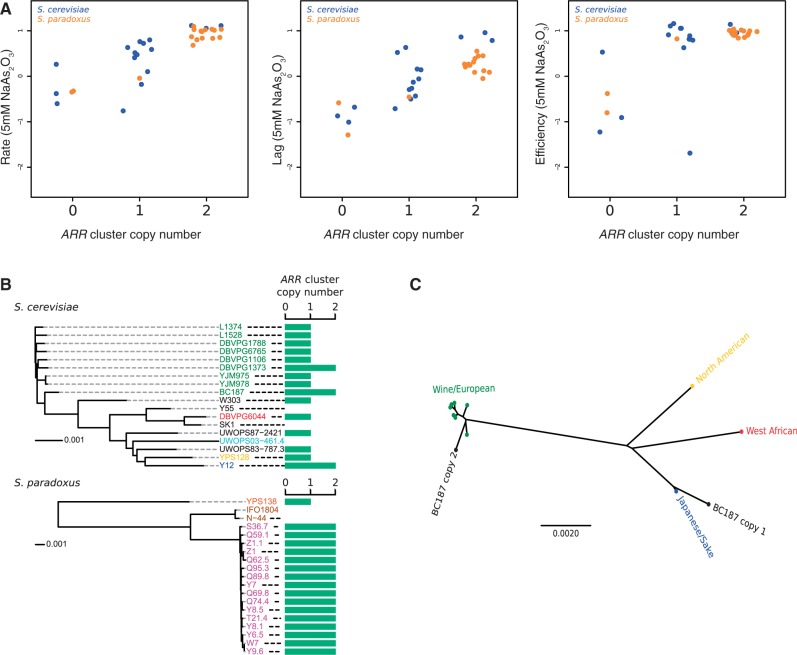
Convergent evolution of *ARR* cluster copy number. (*A*) Growth rate, length of mitotic lag, and mitotic growth efficiency in medium containing 5 mM sodium arsenite oxide for strains with different *ARR* cluster copy number. Units are on a log_2_ scale and relative to the *S. cerevisiae* reference strain derivative BY4741. The strain data points are jittered along the horizontal dimension to increase visibility. (*B*) Distribution of the *ARR* cluster copy number variant within the populations of *S. cerevisiae* and *S. paradoxus*. Strain colors denote subpopulation origin as in figure 2. The strain trees are neighbor-joining trees based on genome-wide SNP distances, and the scale bars indicates sequence distance in units of SNPs per basepair (distance scales differ between the species). (*C*) The two copies of the *ARR* gene cluster in the Wine/European strain BC187 were computationally phased and the sequences of the two copies were clustered with the corresponding sequences from the clean lineage strains of *S. cerevisiae* using the neighbor-joining algorithm. Although the Japanese/Sake strain (Y12) carries two copies, the haplotypes are very similar in sequence and are represented here by a consensus version where the few positions that are polymorphic between the two haplotypes have been masked out. The scale bar indicates sequence distance in units of SNPs per basepair.

One region of the genome exhibiting CNV within both *S. cerevisiae* and *S. paradoxus* is the *ARR* gene cluster, containing three contiguous genes involved in the cellular detoxification of arsenic and antimonyl compounds; the transcription factor *ARR1*, the arsenate reductase *ARR2*, and the plasma membrane transporter *ARR3*. We hypothesized that CNV in this gene cluster would impact growth phenotypes in media containing arsenic compounds and tested this using previously collected phenotype data (Warringer et al. 2011). In both *S. cerevisiae* and *S. paradoxus*, we found a strong association between *ARR* cluster copy number and mitotic growth rate, length of mitotic lag phase and mitotic growth efficiency in the presence of arsenite (fig. 3*A*). In an additive model, *ARR* copy number explains 50% (*P* = 1.5×10^−^^3^) and 92% (*P* = 1.2×10^−^^9^) of the phenotypic variation in growth rate in *S. cerevisiae* and *S. paradoxus,* respectively, and 71% (*P* = 2.5×10^−^^5^) and 82% (*P* = 5.8×10^−^^7^), respectively, of the variation in the length of lag time. Interestingly, for the growth efficiency phenotype, additivity breaks down as there is no difference between strains with one copy and strains with two copies. This result is biologically consistent with a model where the rate at which the cell can expel arsenic compounds increases with the number of *ARR* copies, but the energy cost per arsenite molecule exported is constant and independent of copy number above one.

In *S. paradoxus,* the distribution of the *ARR* gene cluster CNV tracks the population structure, with all strains from the European population having two copies, the North American strain having one copy and the two Far Eastern strains missing the region. The variant distribution within *S. cerevisiae* shows a more complex pattern with evidence for both convergent amplification and introgression between lineages (fig. 3*B*). The region is found in one copy in most strains in the species, missing in the Malaysian strain UWOPS03-461.4 and the predominantly West African mosaic strains SK1 and Y55, and in two copies in the sake strain Y12 and two strains from the Wine/European cluster. By computationally phasing the haplotypes of the two copies of the Wine/European strain BC187, we found that one of these copies clusters phylogenetically with the alleles of the other Wine/European strains as expected, whereas the other clusters with the Y12 copies, indicating that this copy has been introgressed from the sake lineage into parts of the Wine/European population (fig. 3*C*). The other Wine/European strain with a duplication, DBVPG1373, however harbors two copies with very low internal sequence divergence and with no similarity to the sake copies, implying that this is the result of an independent duplication event within the Wine/European population. These findings demonstrate convergent evolution of *ARR* cluster duplication and loss both between different lineages within *S. cerevisiae* and between *S. cerevisiae* and *S. paradoxus.* It is tempting to speculate that the *ARR* cluster CNV has been driven by differences in environmental arsenic concentrations between the habitats of different yeast lineages.

### Derived Alleles in Yeast Tend to Be Deleterious and Private to a Single Population

**Fig. 4.**
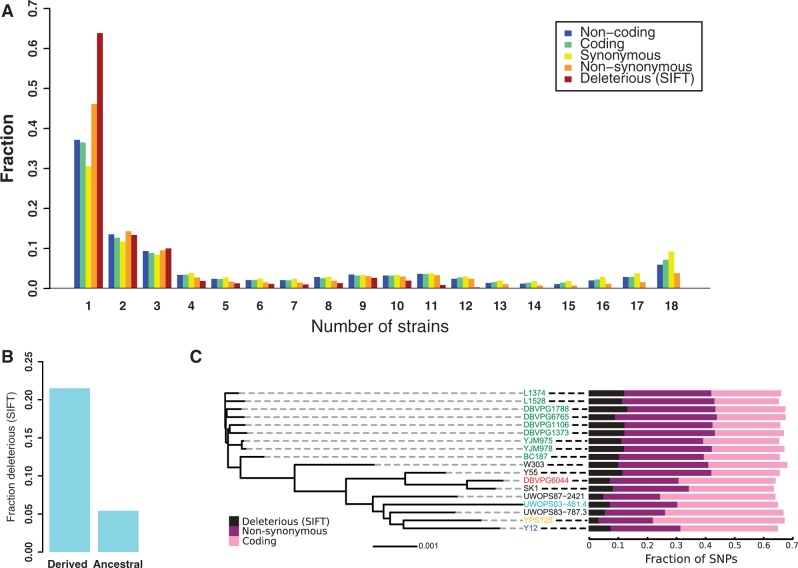
Distribution of SNPs within the *S. cerevisiae* population. (*A*) The derived allele frequency spectrum for SNPs with different coding effects. The ancestral state of each SNP was inferred by using *S. paradoxus* as an outgroup. (*B*) SNP alleles inferred to be derived are much more frequently predicted to be deleterious by SIFT than alleles predicted to be ancestral (21.5% vs. 5.4%, respectively). (*C*) The effect on gene sequences of derived alleles that are found in only a single strain. Strain colors denote subpopulation origin as in figure 2. The strain tree is a neighbor-joining tree based on genome-wide SNP distances and the scale bar indicates sequence distance in units of SNPs per basepair.

To predict the effects of SNPs on protein function, we employed the Sorting Intolerant From Tolerant (SIFT) algorithm, which estimates the probability of an amino acid substitution being deleterious based on evolutionary conservation of the site across a large number of species (Kumar et al. 2009), on all nonsynonymous SNPs identified in the 18 *S. cerevisiae* strains with at least 8× coverage mapped to the reference genome. Using the *S. paradoxus* population as outgroup, we also inferred the most likely ancestral state at each polymorphic locus, allowing the polarization of alleles into ancestral and derived. Overall, we found a strong tendency for derived alleles with higher functional potential to be shifted toward low frequencies (fig. 4*A*). Most derived alleles are present in only one (37%) or a few strains, but among nonsynonymous alleles the bias is more pronounced with 46% occurring in only a single strain. Among nonsynonymous alleles predicted to be deleterious the bias toward lower frequencies is even stronger with 64% occurring in a single strain. These patterns reflect the effects of purifying selection and agree with observations made in the human (Abecasis et al. 2012) and *Arabidopsis thaliana* (Cao et al. 2011) populations. Derived, and therefore recent, alleles are four times more likely to be classified as deleterious than ancestral alleles (fig. 4*B*), consistent with elevated negative selection against them in the species as a whole. We also specifically considered SNPs where the derived allele is private to a single strain and found that such alleles present in Wine/European strains are more frequently nonsynonymous, and the nonsynonymous SNPs are more frequently predicted deleterious than SNPs in other strains (fig. 4*C*). This suggests relaxed negative selection in the Wine/European population, potentially associated with a recent population expansion into a new and beneficial niche such as that introduced by humans with the emergence and spread of wine production over the last 7,000 years (Borneman et al. 2013). Running SIFT in the same fashion on variants identified in the *S. paradoxus* population, we find that derived alleles in this species are on average slightly less often deleterious than derived *S. cerevisiae* alleles (17.8% vs. 21.5%), also consistent with recently relaxed selection in *S. cerevisiae*.

### Loss-of-Function Variants in the *S. cerevisiae* Population

**Fig. 5.**
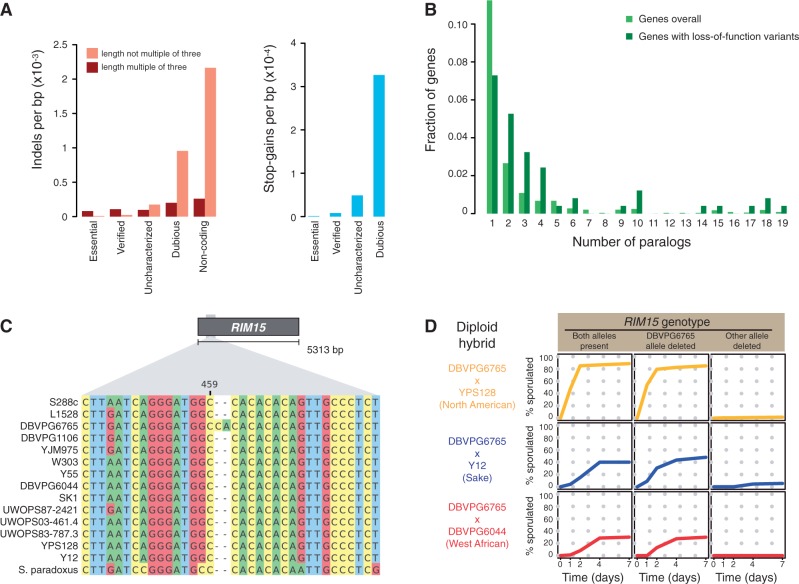
Loss-of-function variants in the *S. cerevisiae* population. (*A*) Frequencies of indels and stop-gain SNPs in different categories of genes. Essential genes refer to genes for which the deletion in the BY reference strain background is not viable. (*B*) The distribution of the number of paralogs for genes with loss-of-function variants and for genes overall. The number of paralogs for each protein coding gene in the *S. cerevisiae* reference genome was estimated as the number of other genes in the genome returning BlastP hits with an e-value < 10^−50^ and with the alignment covering at least 80% of the query protein length. We note that because of CNV the exact number of paralogs for a given gene will vary between strains. The fraction of genes with zero paralogs is omitted. (*C*) A 2-bp insertion in the strain DBVPG6765 disrupts the translational reading frame of the gene *RIM15*. Sequences of *S. cerevisiae* strains and one *S. paradoxus* strain (the reference strain CBS432) for a segment surrounding the insertion in *RIM15* are displayed. (*D*) The phenotypic effect of the frameshifting insertion variant was tested by deleting the *RIM15* gene in the DBVPG6765 strain and in three other strains representing major phylogenetic lineages within *S. cerevisiae*. Diploid hybrids were then constructed between DBVPG6765 and the other three strains, containing alleles of *RIM15* from both parental strains or only from one of them. These diploid strains were tested for their ability to sporulate in KAc medium by scoring the proportion of cells that have undergone sporulation at different time points. In all of the three genetic backgrounds, presence of only the DBVPG6765 *RIM15* allele leads to dramatically lower sporulation efficiency.

Certain classes of variants are expected to have dramatic consequences on gene products and therefore constitute particularly interesting candidates for contributing to phenotypic variation. Taking advantage of the well-annotated reference genome of *S. cerevisiae,* we predicted highly probable loss-of-function variants in the form of prematurely introduced stop codons and frameshifting indels across all 19 *S. cerevisiae* strains. Overall, these variants are enriched toward the 3′-end of ORFs (3.7-fold enrichment in the last 5% of ORFs, *P* < 10^−^^21^). This reflects lower purifying selection pressures against mutations that only perturb translation of the very C-terminal end of the protein and is in line with previous observations (Liti, Carter, et al. 2009; Jelier et al. 2011). Within ORFs, indels with lengths that are multiples of three are highly enriched when compared with noncoding sequence, consistent with strong purifying selection against frameshifts (fig. 5*A*). To test the possibility that full-length proteins could be produced from frameshifted genes by programmed translational frameshifting, we searched for sequence signatures believed to mediate this process (Theis et al. 2008) but found no evidence for this. As a group, ORFs classified as dubious do not display the strong signs of purifying selection described above, implying that most of these are unlikely to constitute functional genes.

Excluding ORFs classified as dubious and variants positioned in the very 3′ end of genes (last 2% of the ORF), we identified 242 genes harboring loss-of-function variants in at least one *S. cerevisiae* strain. This set is enriched for genes classified as functionally uncharacterized (3.1-fold enrichment, *P* < 10^−^^33^), demonstrating that this group of genes on average are under lower purifying selection pressures (fig. 5*A*). One explanation for this result could be that there is a bias in the investigation, and therefore also in the functional classification, of yeast genes toward genes that are more phenotypically important and therefore also under stronger selection. Genes with putative loss-of-function variants are also less likely to be essential in the reference strain S288c (11-fold depletion, *P* < 10^−^^15^). Only four essential genes with loss-of-function variation were found. In all these cases, the variants were positioned either very late in the reading frame (*PZF1*) or very early with an alternative start codon nearby (*CBF2*, *LTO1*), or the affected gene overlapped an essential gene so that the essentiality classification is likely to be false (*YJR012C*; Ben-Shitrit et al. 2012).

Subtelomeric genes are overrepresented among genes harboring loss-of-function variants (3.5-fold enrichment, *P* < 10^−^^17^), consistent with the idea that the subtelomeres are evolutionary dynamic regions for which selection pressures vary over time and space. Genes that are part of multicopy gene families show a similar higher tendency to harbor loss-of-function mutations (fig. 5*B*). This observation has also been made in humans (MacArthur et al. 2012) and is consistent with a model where these mutations can sometimes escape the effects of negative selection due to functional redundancy with a paralog elsewhere in the genome. However, it is also consistent with a model where the kinds of genes that tend to have paralogs are simply under lower or more variable selection pressures in general (Woods et al. 2013). Genes with loss-of-function mutations are enriched for gene ontology terms related to transmembrane transport of small molecules and ions as well as flocculation. These genes also have on average a lower number of gene ontology terms annotated to them (1.89 vs. 2.5 for the whole genome for the “biological process” domain, *P* = 1.1×10^−^^5^, excluding genes with zero terms), indicating a lower degree of pleiotropy.

We reasoned that if the predicted loss-of-function variants are truly disrupting the function of the affected genes, then we should see evidence of lower purifying selection acting on these genes, in general, within *S. cerevisiae*. Indeed, genes harboring such variants have higher ratios of nonsynonymous over synonymous polymorphisms (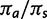) within the species than genes without loss-of-function variants (0.643 vs. 0.485, respectively, *P* < 10^−^^15^ Fisher’s exact test). To test whether this relaxed selection pressure is specific to *S. cerevisiae* or reflects a trend that has been running over longer evolutionary timescales, we also evaluated the observed correlation in *S. paradoxus*. Overall, *S. paradoxus* orthologs of *S. cerevisiae* genes with predicted loss-of-function variants have higher 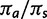 ratios than other *S. paradoxus* genes (0.555 vs. 0.457, respectively, *P* < 10^−^^13^ Fisher’s exact test). Thus, the selection acting on these genes has been generally low at least over the more than 2 billion generations back to the most recent common ancestor of *S. cerevisiae* and *S. paradoxus* (Dujon 2010). To test whether the emergence of loss-of-function mutations in certain *S. cerevisiae* strains is associated with a further relaxation of selection specifically in these lineages, we compared 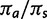 ratios between those strains carrying the loss-of-function variant of a gene and those strains with the intact ORF and found no difference (0.642 vs. 0.639, *P* = 0.8774, Fisher’s exact test). Together, these results are largely consistent with a nearly neutral model of gene evolution where the strength of purifying selection is to a large extent a conserved property of the particular gene.

As a case study for the phenotypic implications of loss-of-function variation in natural yeast populations we focused on the gene *RIM15*, in which we identified a frameshifting 2 bp insertion private to the Wine/European strain DBVPG6765 (fig. 5*C*). We also observed selection against the DBVPG6765 allele in the genomic region containing *RIM15* during the generation of a four-parent advanced intercross line (Cubillos et al. 2013). *RIM15* encodes a protein kinase believed to be involved in the regulation of cell division, proliferation, and sporulation in response to nutrient availability (Broach 2012). To test whether the identified frameshift variant impairs these cellular processes, we constructed diploid hybrids between DBVPG6765 and three representatives of other major *S. cerevisiae* lineages. We deleted either of the two *RIM15* copies to obtain reciprocal hemizygote strains that contain either only the DBVPG6765 allele or only an allele with an intact reading frame. We find that the frameshifted DBVPG6765 allele has a massive negative impact on the ability of the cell to undergo meiosis and form spores in response to nutrient starvation (fig. 5*D*). Thus, the strain DBVPG6765 harbors a nonfunctional *RIM15* allele despite its strongly detrimental effects on traits considered to be highly related to fitness. We also find that *RIM15* has very low expression level in this strain in a RNA-seq data set (Skelly et al. 2013). Interestingly, an independent loss-of-function variant in *RIM15* has been described in sake yeasts, where it is linked to a reduced ability to enter quiescence and an associated increased rate of ethanol production (Watanabe et al. 2012). Further work is needed to elucidate why this strain of *S. cerevisiae* carries this highly deleterious variant.

## Conclusions

We found surprising and striking differences in the nature of genomic diversity within the two yeast species of *S. cerevisiae* and *S. paradoxus*. Despite lower levels of genetic divergence between strains, *S. cerevisiae* displayed greater diversity in the presence and absence as well as copy number of genetic material than did *S. paradoxus*. Our results strongly reinforce that the subtelomeres are the major focal regions for functional evolution; they are almost exclusively the sites for structural, gene content and CNV and are also highly enriched for loss-of-function variants. The relevance of this subtelomeric diversity to phenotypic variation is underscored by the finding that a third of QTLs for ecologically relevant traits map to the subtelomeric regions (Cubillos et al. 2011), even though they only constitute approximately 8% of the genome. The complex structure of the subtelomeres unfortunately also makes them the most problematic regions of the genome to assemble and analyze, hampering nucleotide-level dissections of this variation and its functional consequences. Promising avenues for overcoming this technical limitation of short-read sequencing include the subcloning of individual subtelomeres, allowing independent sequencing and assembly, and the use of emerging sequencing technologies that produce much longer reads (Bashir et al. 2012; Loomis et al. 2013). We find systematic trends in the types of genes that tend to be affected by certain types of potentially functional variation. Genes displaying copy number and loss-of-function variation as well as genes not present in the reference genome are enriched for functions related to interaction with the external environment, for example, sugar transport and metabolism, flocculation and cell adhesion, and metal transport and metabolism. It is plausible that this reflects variation in the environmental conditions of different strain habitats, leading to selective pressures for these cellular functions that vary across time and space and resulting in either gain or loss of gene functions in different lineages. The strong population structure of natural yeast strains has a critical influence on virtually all aspects of genomic diversity, and we stress the importance of considering this in analysis and interpretation of results. Finally, our results demonstrate the high utility of short-read next-generation sequencing for yeast population genomics, especially the value of de novo assembly to identify variation in genome content, and we hope that the sequence data and assemblies presented will be useful to the yeast community as well as the broader evolutionary and population genetics and genomics communities.

## Materials and Methods

### Genome Sequencing and De Novo Assembly

Strains were selected for whole-genome sequencing to encompass most of the genetic diversity of *S. cerevisiae* and *S. paradoxus*; at least one strain representing each major phylogenetic lineage as defined in Liti, Carter, et al. (2009) (in *S. cerevisiae,* the North American, Sake/Japanese, West African, Wine/European, and Malaysian lineages; in *S. paradoxus,* the far Eastern, American, and European lineages) as well as a larger number of strains from the European populations of both species. Additionally, in *S. cerevisiae*, we selected the mosaic strains W303, SK1, and Y55, which are popular laboratory strains as well as two wild mosaic strains isolated from the Bahamas and Hawaii, that phylogenetically cluster closer to the non-European lineages than most other mosaic strains. 

DNA was extracted using the phenol chloroform protocol. Samples were sequenced using the Illumina GAII and HiSeq platforms, with 2× 108 bp or 2× 100 bp paired end libraries prepared as previously described (Parts et al. 2011). SGA (Simpson and Durbin 2012) was used to perform de novo assembly of all strains that had sequencing coverage of 20× or higher after quality filtering (by the SGA preprocess program), including scaffolding of contigs using Illumina paired-end information. Reads were error corrected with a k-mer length of 41 and a minimum of five read pairs were required to link two contigs into a scaffold. Contigs/scaffolds smaller than 200 bp were discarded. Further scaffolding was then performed using low-coverage Sanger capillary data previously produced for the same strains (Liti, Carter, et al. 2009). The paired-end Sanger reads were mapped onto the SGA scaffolds using SSAHA2 (Ning et al. 2001), and scaffold pairs connected by at least two read pairs in which both reads had a mapping quality of 254 were used as input for the stand-alone scaffolder SSPACE (Boetzer et al. 2011), which was run without contig extension and a maximum allowed deviation from the mean pair distance of 50%. Mean pair distances were estimated for each strain individually from pairs where both reads mapped within the same SGA scaffold. To avoid incorrect scaffolding because of collapsed repeats in assemblies, scaffold ends showing signs of collapse in the form of higher Illumina coverage were excluded from Sanger scaffolding. The Illumina reads were mapped to the SGA assemblies using BWA 0.6.1 (Li and Durbin 2009) with the “−q 10” parameter and scaffold edges where the log_2_-ratio between the average coverage of the outermost 5 kb and the genome-wide median coverage was higher than 0.5 were excluded. After scaffolding with the Sanger reads, the SGA gapfill program was run to fill in some of the resulting gaps using the error-corrected Illumina reads. For four of the *S. paradoxus* strains assembled (Y8.5, Y9.6, Z1, and W7), no Sanger reads are available, and so further scaffolding could not be performed for these strains. Genome sizes reported in the text do not take the large ribosomal DNA (rDNA) tandem repeat array on chromosome XII into account, which in the S288c reference genome is represented by two copies (Johnston et al. 1997) and which collapses into a single copy in our de novo assemblies. We also note that the mitochondrial genomes did not assemble well, likely a result of the very high AT content.

Contaminant sequences displaying high (∼99%) identity to genomes from the prokaryotic *Staphylococcus* genus were identified in the assembly of the strain DBVPG6044. All contigs/scaffolds that had a BlastN match to a *Staphylococcus* species in the top five hits from the NCBI nr database were removed from the assembly. No hybrid contigs or scaffolds containing both *Saccharomyces* and *Staphylococcus* were found.

### Assembly Scaffolding Using Genetic Linkage Data

Four of the *S. cerevisiae* strains have been used as parents for advanced intercross lines as part of projects to study complex traits and recombination, and the genomes of a large number of segregants from these lines have been sequenced: 192 F12 segregants from a two-parent cross between YPS128 and DBVPG6044 (Illingworth et al. 2013) and 192 F12 segregants from a four-parent cross between YPS128, DBVPG6044, Y12, and DBVPG6765 (Cubillos et al. 2013); in both cases sequenced by paired-end Illumina technology with 100-bp read lengths. The patterns of linkage disequilibrium (LD) between SNPs in these artificial populations were used to further scaffold the de novo assemblies of the four parental strains. First, for each of the parental genome assemblies, the Illumina reads for the other parental strains were mapped to the assembly and SNPs were called (read mapping and SNP calling performed as described in the section “Read-mapping and variant calling”). For the two strains of the two-parent cross (YPS128 and DBVPG6044), only data from this cross was used, and data from the four-parent cross was used only for the remaining two strains (Y12 and DBVPG6765). The segregant individuals were then genotyped at the resulting lists of SNPs using samtools mpileup 0.1.18 (Li et al. 2009), assigning the corresponding allele if the log-likelihood ratio between the homozygous states of the two alleles was bigger than 10 or smaller than −10, assigning unknown genotype if in-between. Segregant individuals showing signs of diploidy or DNA contamination as assessed by genome-wide patterns of heterozygosity were excluded (20 out of 192 individuals in the two-parent cross and 15 out of 192 in the four-parent cross). Using the resulting genotype calls, LD in units of *r*^2^ was then computed between all pairs of SNPs using PLINK (Purcell et al. 2007). A model for the approximation of physical base-pair distances from genetic distances of the form *r*^2 ^= *e*^−^*^λ^^d^*, where *d* is physical distance between two SNPs in units of base pairs and *λ* is a constant, was fit by nonlinear least squares to the set of values from SNPs located within the same scaffold (only scaffolds of size 50 kb and bigger were used for fitting). LD between SNPs on different scaffolds was used to construct pairwise scaffold–scaffold links, with relative orientations inferred by comparing the strength of LD in the four corner elements of the matrix of LD values between all SNPs in the two scaffolds. The pairwise scaffold–scaffold links then constitutes a directed graph through which each linkage group should be traceable as a simple path. The paths were traced partly using the scaffolding algorithm of SGA and partly by manual curation aided by visualizations in Cytoscape (Shannon et al. 2003). Scaffolds that could be positioned but not reliably oriented because of a lack of difference in LD profiles between the two ends or because they did not have at least two SNPs were not included in the linkage group scaffolds but left as unplaced.

### Genome Assembly Validation

Diagnostic PCR was used to confirm the absence and presence of genomic regions identified as variable between strains in the de novo assemblies. Primers were designed to target 14 different regions in all 14 strains of *S. cerevisiae* with de novo assemblies plus the S288c reference strain and for 9 of the regions also in all of the 13 *S. paradoxus* strains with de novo assemblies (supplementary fig. S3, Supplementary Material online). Different primers were designed for the two different species and placed in nonpolymorphic locations. Primers are listed in supplementary table S1, Supplementary Material online.

As an additional validation, the de novo genome assembly of the *S. cerevisiae* strain SK1 was compared with another recently released assembly of this strain (van Overbeek et al. http://cbio.mskcc.org/public/SK1_MvO/ [last accessed October 4, 2013]; Sasaki et al. 2013), produced primarily from 454 pyrosequencing reads and with additional error-correction and gap closing efforts. The assemblies were compared to identify any false negatives in the sense of sequence incorrectly left out of our assembly and false positives in the sense of sequence or assembly artifacts in our assembly that do not represent actual sequence present in the genome of this strain. A single region larger than 1 kb was found to be present in the van Overbeek et al. assembly and absent in ours. Inspection revealed that this is a technical cloning vector that has not been introduced into the SK1 derivative sequenced here, and thus does not represent genuinely missing biological sequence. Thirty-one regions larger than 1 kb were present in our assembly and absent in the van Overbeek et al. assembly. To test whether these are assembly artifacts in our assembly or sequence incorrectly left out of the van Overbeek et al. assembly, the 454 reads used to construct the van Overbeek et al. assembly were mapped back to our SK1 assembly using SSAHA2 (Ning et al. 2001), and the depth of coverage was assayed in these 31 regions. Excluding the 100 bp at the edge of contigs, all positions in these regions were covered by five or more reads with mapping qualities of at least 75. This shows that these sequences are actually present in the strain SK1 but have for some technical reason been left out of the van Overbeek et al. assembly. No false positives and no false negatives of this kind were thus identified in our SK1 de novo assembly.

### Reference Genomes and Annotation Data Used

For *S. cerevisiae*, the S288c reference genome and corresponding annotation files (Release R64-1-1, downloaded from the *Saccharomyces* Genome Database [http://yeastgenome.org/, last accessed January 28, 2014] on February 5, 2011) was used for reference-based analyses. The sequence of the 2-µm circle plasmid was downloaded from NCBI’s GenBank (accession NC_001398). For *S. paradoxus*, the CBS432 reference genome from Liti, Carter, et al. (2009) was used, with annotation being obtained from Scannell et al. (2011). A *S. paradoxus* CBS432 mitochondrial sequence was obtained from Prochazka et al. (2012) (GenBank accession: JQ862335.1). For analyses assaying the presence of genomic material in the *S. paradoxus* reference genome, an alternative version of the CBS432 assembly that includes some additional sequence that was incorrectly left out from the original assembly was used (available from Liti, Carter, et al. [2009]). We define the subtelomeric regions as the outermost 33 kb of each reference chromosome, following Brown et al. (2010). Annotation on the essentiality of genes was that of the *S. cerevisiae* reference strain S288c.

### Identification of Genome Content Differences

To identify genomic material present in a given genome but absent in another, alignments were constructed between genome assemblies using BlastN without low-complexity filtering, and a region in the query sequence was called as not present in the target sequence if no alignment of either length 100 bp and sequence identity of 75% or length 50 bp and sequence identity 90% was found. For the reported results only such identified regions of a minimum length of 1,000 bp were considered.

### Genome Assembly Annotation

The protein sequences of all nuclear ORFs annotated as genuine genes in the reference genomes of *S. cerevisiae* and *S. paradoxus*, respectively (in the former species, all ORFs not classified as “dubious” and in the latter all ORFs classified as “real”) were searched for in the de novo assemblies using exonerate version 2.31.12 (Slater and Birney 2005) with the “protein2dna” alignment model. For each reference protein query, the most likely homologous gene in each strain genome was inferred by sequence similarity and synteny comparisons from the set of all candidate alignments with more than 90% sequence similarity to the query and with an exonerate alignment score within 90% of the top scoring alignment for the gene. This was done by identifying and selecting in priority order: top-scoring alignments supported by gene synteny to the reference genome on both sides, top-scoring alignments supported by synteny on one side and being right next to the edge of the scaffold on the other side, and nontop scoring alignments supported by synteny on both sides. If more than one reference protein query had their inferred homolog localized to the same part of the assembly (both start and end positions differing by less than 100 bp), only one of them was kept (prioritizing perfect synteny support combined with being the top scoring alignment, then higher alignment score, and then longer gene length).

Ab initio gene prediction for genes not present in the reference genome was performed using GeneMarkS version 4.10d (Besemer et al. 2001) in the self-training mode and with the “−euk” option for intronless eukaryotic gene prediction. Predicted genes that did not overlap the coordinates of the inferred reference genome homologs and that additionally, to account for potential reference homologs missed by the above homology inference, did not display high similarity to any reference genome gene (defined as the presence of a BlastN hit covering either 80% of the query length or 200 bp and with a sequence similarity of at least 90%) were classified as nonreference genes. For the numbers reported, only predicted genes with lengths of 300 bp or more were included.

### Read-Mapping and Variant Calling

For purposes of identification of SNPs and short indels, reads cleaned from adapter contamination using cutadapt (http://code.google.com/p/cutadapt/, last accessed January 11, 2011) were mapped to reference genomes and de novo assemblies using Stampy 1.0.18 (Lunter and Goodson 2011) with the “sensitive” parameter, in hybrid mode with BWA 0.5.9 and with the “−q 10” BWA parameter. Nonprimary alignments and nonproperly paired reads were filtered out and duplicate reads were removed using Picard (http://picard.sourceforge.net/, last accessed October 22, 2012). Before SNP calling, reads were locally realigned using SRMA 0.1.15 (Homer and Nelson 2010) and read base qualities were capped by their Base Alignment Qualities (Li 2011) as computed by samtools 0.1.18 (Li et al. 2009). SNPs were called on the read alignments using FreeBayes 0.9.5 (http://bioinformatics.bc.edu/marthlab/wiki/index.php/FreeBayes, last accessed May 2, 2012) set for haploid samples. Short indels were identified using Dindel 1.01 (Albers et al. 2011) executed in pooled mode. Individual indel genotype calls for each haploid strain were obtained by extracting the genotype likelihoods as computed assuming diploid samples and assigning the corresponding allele if the log-likelihood ratio between the homozygous states of the two alleles was bigger than 5.3 or smaller than −5.3, assigning unknown genotype if in-between. Only indels shorter than 60 bp were called. Indels were not counted as affecting the ORF of a gene if the equivalent indel region (Krawitz et al. 2010) was not completely contained within the ORF.

### Copy Number Variation

CNV was identified by mapping the Illumina reads to the *S. cerevisiae* and *S. paradoxus* reference genomes. The average depth of read coverage was computed in nonoverlapping windows of size 500 bp and normalized by the genome-wide median coverage for each strain, and the log_2_ values of these ratios were then plotted. Regions of the genome showing coverage variation between strains were identified manually by systematically inspecting the plots. Regions annotated with transposable element associated features were masked out at the plotting level and excluded from the analysis. As the *S. paradoxus* reference genome is not comprehensively annotated for transposable elements, masking was applied to regions of the genome displaying high sequence similarity to any *S. cerevisiae* transposon-associated feature sequences (BlastN e-value <10^−^^25^). One strain of *S. cerevisiae* (YJM981) and four strains of *S. paradoxus* (KPN3829, Q31.4, Q32.3, UFRJ50816) were excluded from copy number analysis because they had a coverage of reads mapped to the reference genome of below 8×. For the gene family-based CNV analysis, read depth was aggregated across all members of a gene family and normalized by the genome-wide median coverage as above. The gene families used are those defined in Christiaens et al. (2012). Although the true amount of CNV in *S. paradoxus* is likely slightly underestimated due to the presence of gaps in the reference genomes, this underestimation is unlikely to explain the large difference in the amount of CNV observed between *S. cerevisiae* and *S. paradoxus*. 9% of subtelomeric sequence (and 1.5% of the whole genome) in the *S. paradoxus* reference assembly consists of gaps—assuming very conservatively that all of this gapped subtelomeric sequence harbors CNV the estimate for the total size of genomic regions containing CNVs would increase from 142 to 232 kb (and to 319 kb extending the assumption to all gapped sequence in the whole genome), which is still considerably smaller than the 423 kb in *S. cerevisiae*. 

### *ARR* Gene Cluster Analysis

The haplotypes of the two *ARR* gene copy clusters in the strain BC187 were phased by first calling SNPs in a 6,400-bp region encompassing the cluster using samtools mpileup on mapped Illumina reads, and then phasing the SNPs using the ReadBackedPhasing program from the Genome Analysis Toolkit (McKenna et al. 2010) with both Illumina and Sanger paired-end reads as input. For the strains Y12 and DBVPG1373, the two *ARR* cluster copies were not sufficiently diverged for phasing to be possible. Neighbor-joining trees were constructed by Seaview (Gouy et al. 2010), and for Y12 a single consensus sequence for the two haplotypes was constructed for phylogenetic analysis by excluding the positions where the two copies differ in sequence (five positions). Data from all the strains on the mitotic growth rate, length of mitotic lag, and mitotic growth efficiency in a medium containing 5 mM sodium arsenite oxide was obtained from Warringer et al. (2011).

### Gene Ontology Enrichment

Gene ontology enrichment analyses were performed at YeastMine (http://yeastmine.yeastgenome.org/, last accessed February 6, 2013) with the Benjamini–Hochberg correction for multiple testing and a *P*-value threshold of 0.05. As gene ontology annotation is not directly available for *S. paradoxus*, genes were mapped to their *S. cerevisiae* orthologs and analyses were performed on the resulting *S. cerevisiae* gene sets. Ortholog mappings were obtained from Scannell et al. (2011).

### *RIM15* Phenotyping

*RIM15* reciprocal hemizygosity strains were constructed by one-step PCR deletion with URA3 as a selectable marker (Salinas et al. 2012). The gene was deleted in the haploid versions of the four parental strains (either *Mat a, ho::HygMX, ura3::KanMX* or *Mat a, ho::NatMX, ura3::KanMX)* and deletions were confirmed by PCR. Strains of opposite mating type were crossed to generate the hemizygotic hybrid diploid strains. Sporulation efficiency was then measured as follows. Strains were grown in 50 ml of YPG (2% peptone, 1% yeast extract, 3% glycerol, and 0.1% glucose) overnight, washed with water three times, transferred to 50 ml of 2% potassium acetate and incubated with shaking at 23 °C. Samples were taken at 1, 2, 4, and 7 days after the start of incubation, and in each sample, the number of cells having formed asci was counted using an optical microscope. Two-hundred cells were assayed for each sample.

## Supplementary Material

Supplementary figures S1–S6 and tables S1 are available at *Molecular Biology and Evolution* online (http://www.mbe.oxfordjournals.org/). 

Supplementary Data
