# Supplementary material for: A High-Definition View of Functional Genetic Variation from Natural Yeast Genomes
Source: Mol Biol Evol. 2014 Jan 14;31(4):872–88. doi: 10.1093/molbev/msu037 (PMC3969562; doi:10.1093/molbev/msu037)
Supplement: Supplementary Data [file supp_31_4_872__index.html]

A high-definition view of functional genetic variation from natural yeast genomes — A High-Definition View of Functional Genetic Variation from Natural Yeast Genomes — A High-Definition View of Functional Genetic Variation from Natural Yeast Genomes — Supplementary Data 

# A High-Definition View of Functional Genetic Variation from Natural Yeast Genomes

## Supplementary Data

files

**Files in this Data Supplement:**

- Supplementary Data - pdf file
- Supplementary Data - pdf file
