# Supplementary material for: A High-Definition View of Functional Genetic Variation from Natural Yeast Genomes
Source: Mol Biol Evol. 2014 Jan 14;31(4):872–88. doi: 10.1093/molbev/msu037 (PMC3969562; doi:10.1093/molbev/msu037)
Supplement: Supplementary Data [file supp_msu037_Supplementary_figures.pdf]

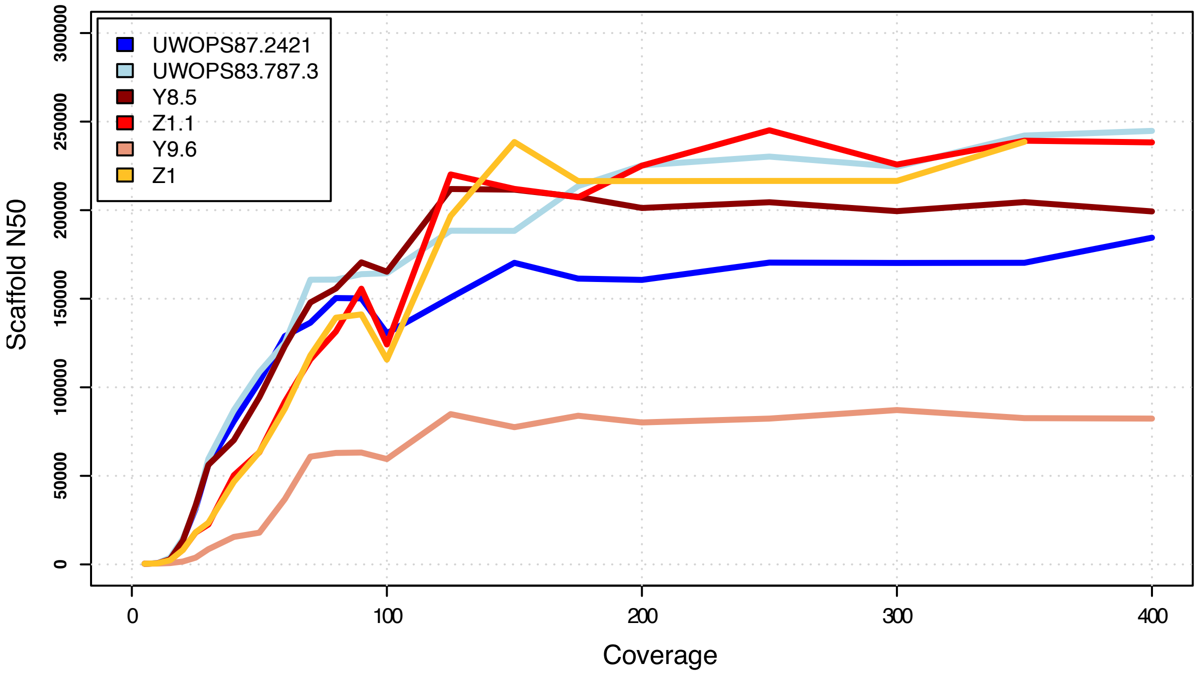

Figure S1. De-novo assembly continuity with varying levels of sequencing coverage. Six strains, two S. cerevisiae (UWOPS87-2421, UWOPS83-787.3) and four S. paradoxus (Y8.5, Z1.1, Y9.6, Z1), were sequenced to very high coverage using Illumina HiSeq technology. The read data sets were randomly downsampled to varying levels of coverage and assembled by SGA, including scaffolding of contigs using Illumina paired-end information. Program parameters were kept constant except for the minimum overlap parameter (the "-m" option to the SGA "assemble"subprogram), which was gradually increased with increasing coverage and the k-mer frequency threshold for error correction (the "-x" option to the SGA "correct" subprogram), which was learned from the data for coverage levels below 100x and set to 5 otherwise.
The values on the horizontal axis refer to raw levels of read coverage, before any filtering.

S. cerevisiae

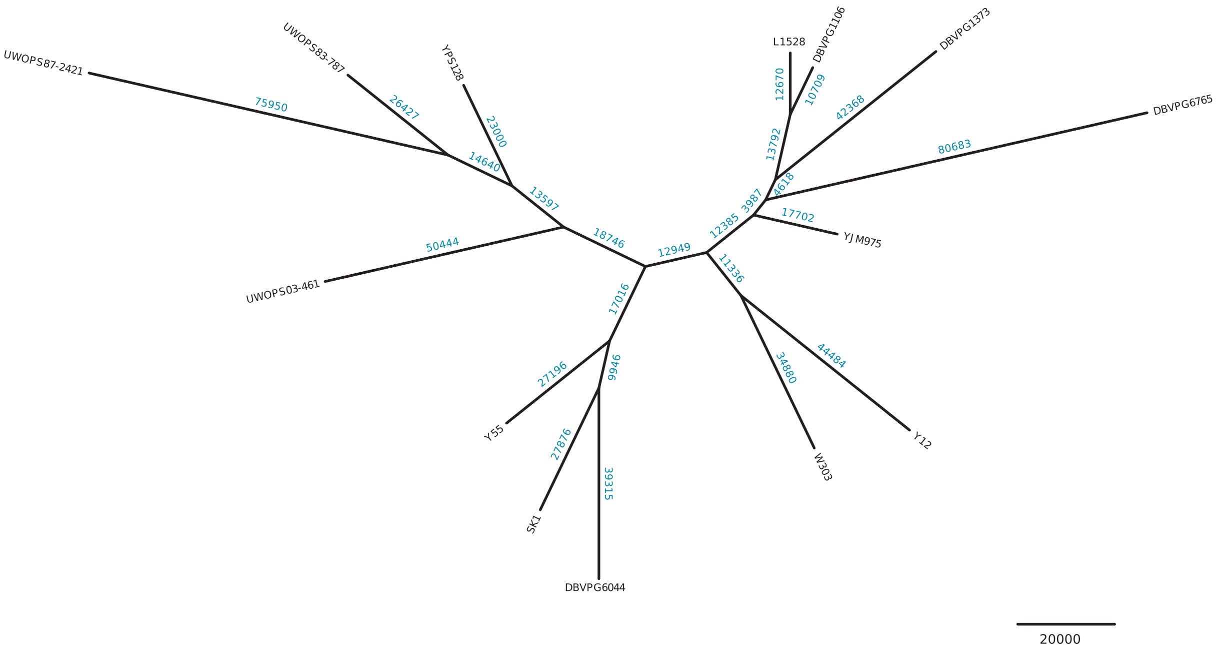

S. paradoxus

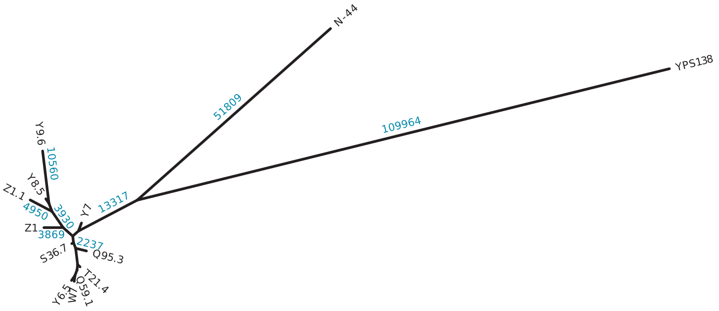

Figure S2. Strains clustered based on genome content differences.
A distance between a pair of strains was set to the amount of genomic material being present in one of the strains and absent in the other (as in figure 2A) and strains were then clustered by the neighbor-joining algorithm as implemented in the APE package (Paradis et al. 2004).

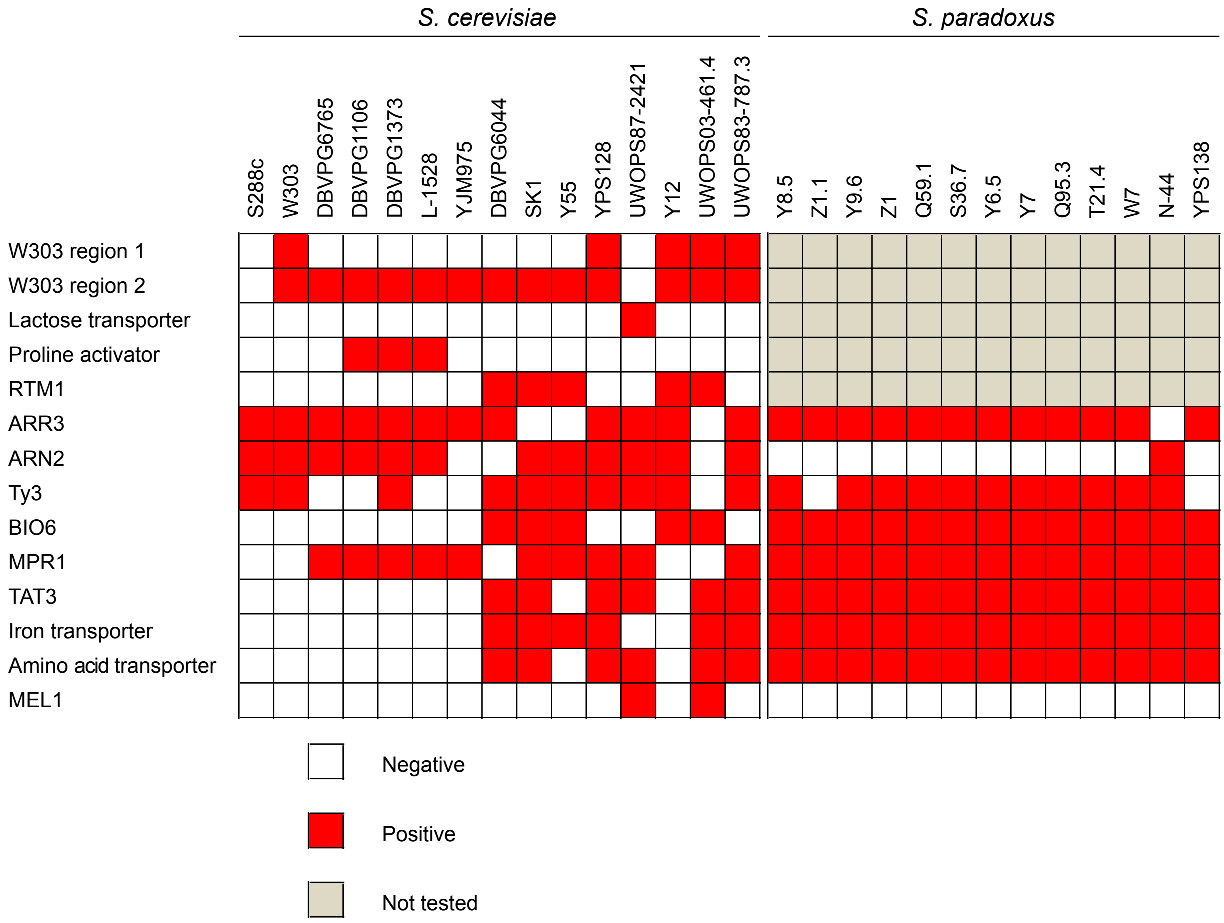

Figure S3. Variable genomic regions in *S. cerevisiae* and *S. paradoxus* strains validated by PCR.
The top five regions are not present in any of the *S. paradoxus* strains and therefore could not be reliably tested by PCR. Regions are named by previously existing gene names or by putative function determined by homology searches. Two regions designated as W303 region 1 and W303 region 2 are variable between W303 and the *S. cerevisiae* reference strain S288. An ORF with homology to a lactose transporter appear to be extremely rare in other *S. cerevisiae* strain genomes (only found in the bioethanol strain JAY291) and otherwise has the best match in *Debaryomyces hansenii*. Rare regions are likely to have originated from horizontal gene transfer, as is the case for the proline activator which is polymorphic within the Wine/European cluster and previously reported to come from a far related yeast species (Novo et al. 2009). Furthermore, several accessory genes like *BIO6, MPR1*, Iron transporter, Amino acid transporter and *TAT3* appear to be stable in *S. paradoxus* and present in all the strains in this species. These genes also appear to be present in other less related *Saccharomyces sensu stricto* species and therefore their patterns of occurrence are consistent with a scenario of multiple independent losses occuring in *S. cerevisiae* lineages. The *S. paradoxus MEL1* primers were designed using the sequence X95505.1 present in Genbank. Overall, this analysis reinforces the finding that there is less variation within *S. paradoxus* than within *S. cerevisiae* in terms of presence and absence of accessory genes.

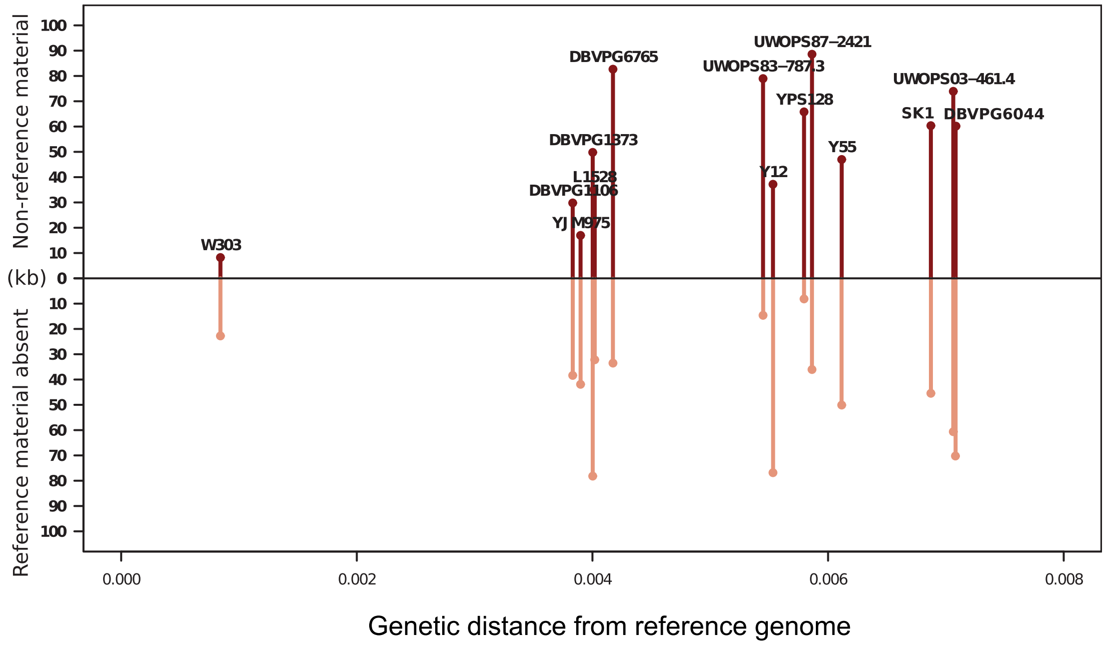

Figure S4. Genome content differences to the *S. cerevisiae* reference genome.
The relationship between genetic distance from the *S. cerevisiae* reference genome as measured in single-nucleotide polymorphisms and the amount of genomic material being present as well as absent in the genome of a given strain. The upper part of the figure displays the amount of genomic material present in a given strain and absent in the S288c reference genome and the lower part the amount of material present in the reference genome but absent in the strain.

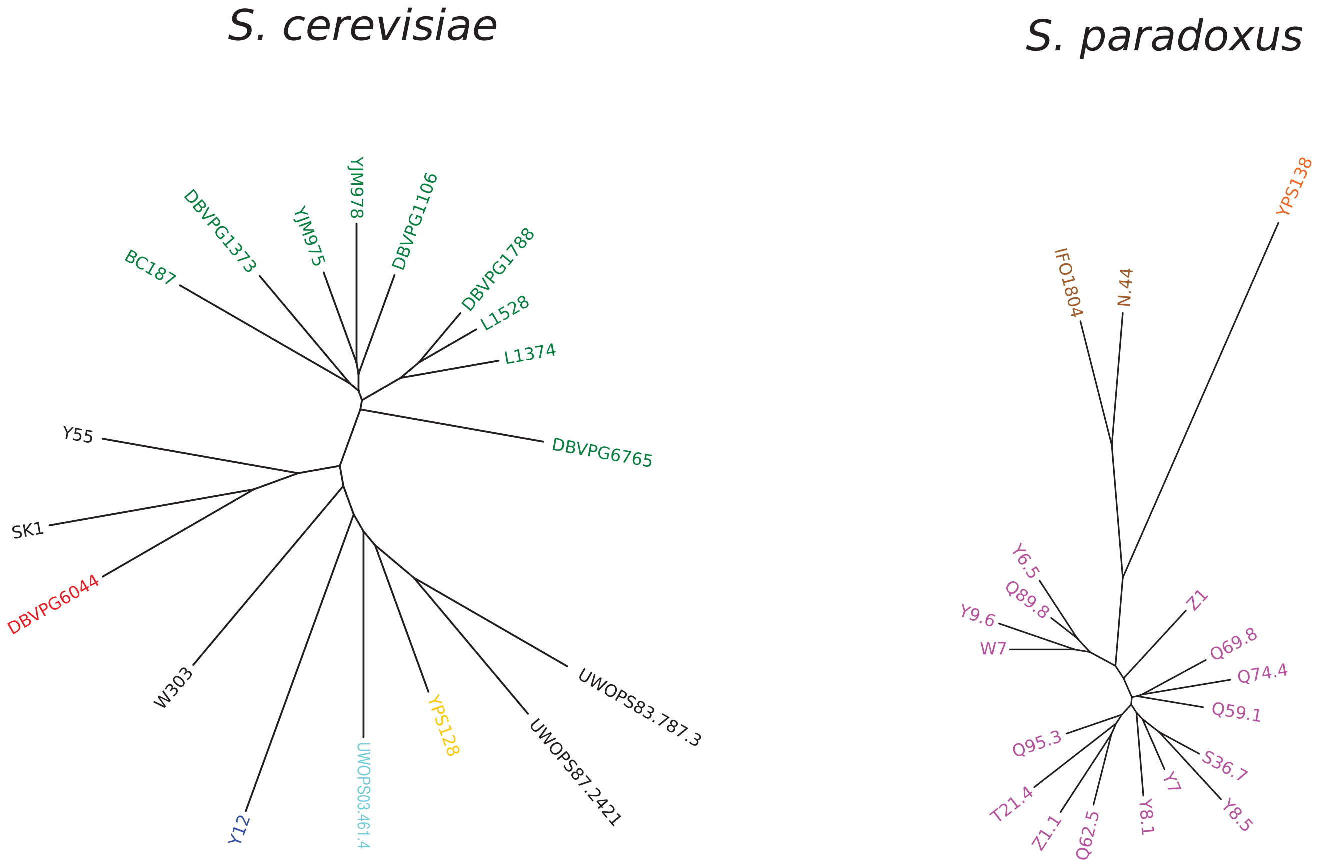

Figure S5. Clustering strains based on copy number variation.
Strains were clustered using the neighbor-joining algorithm on the Euclidian distances between the normalized average coverage values across all regions called as containing copy number variation within the species. The two trees are drawn to the same scale. Strain colors denote subpopulation origin as in figure 2B.

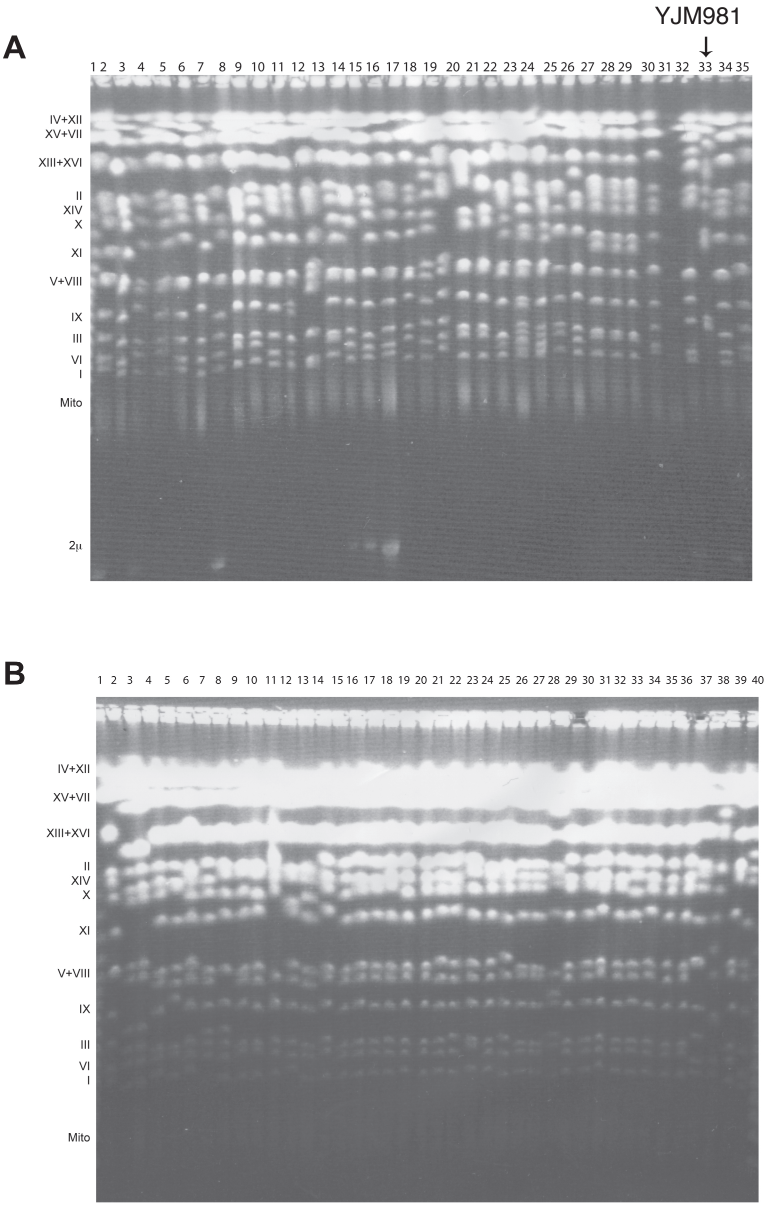

Figure S6. Electrophoretic karyotypes of *S. cerevisiae* and *S. paradoxus* strains.
All strains surveyed in (Liti et al. 2009a) are included. The band patterns reflect chromosome sizes. YJM981 (gel A, lane 33) presents an unconventional karyotype with many of the bands shifted up, indicating increases in size. Chromosome order, on the left side, refers to the S288c reference strain.

Order of samples:
A: *S. cerevisiae*: 1) S288c 2) DBVPG6765 3) SK1 4) DBVPG6044 5) DBVPG1788 6) DBVPG1373 7) DBVPG1853 8) Y55 9) YPS128 10) DBVPG1106 11) DBVPG6040 12) YIIc17_E5 13) YPS606 14) L1374 15) L1528 16) BC187 17) NCYC110 18) NCYC361 19) K11 20) Y9 21) Y12 22) YS2 23) YS4 24) YS9 25) UWOPS83-787.3 26) UWOPS87-2421 27) UWOPS03-461.4 28) UWOPS05-217.3 29) UWOPS05-227.2 30) W303 31) 322134S 32) YJM978 33) YJM981 34) YJM975 35) S288c

B: *S. paradoxus*: 1) S288c 2) UFRJ50791 3) UFRJ50816 4) N-17 5) DBVPG4650 6) DBVPG6304 7) T21.4 8) N-43 9) N-44 10) N-45 11) CBS5829 12) YPS138 13) IFO1804 14) CBS432 15) Y7 16) Q32.3 17) Q59.1 18) Q95.3 19) S36.7 20) Z1.1 21) Y6.5 22) Q62.5 23) Q89.8 24) A4 25) A12 26) KPN3828 27) KPN3829 28) UWOPS91-917.1 29) Y9.6 30) Q74.4 31) Q69.8 32) W7 33) Q31.4 34) Y8.5 35) Z1 36) Y8.1 37) S288c 38) 322134S 39) 378604X 40) 273614N
