# Supplementary material for: A High-Definition View of Functional Genetic Variation from Natural Yeast Genomes
Source: Mol Biol Evol. 2014 Jan 14;31(4):872–88. doi: 10.1093/molbev/msu037 (PMC3969562; doi:10.1093/molbev/msu037)
Supplement: Supplementary Data [file supp_msu037_Supplementary_table_1.pdf]

Supplementary table 1. Primers used in the diagnostic PCR of the variable regions reported in Figure S3.

Sequence	Primer forward	Primer reverse
S. cerevisiae		
W303 region 1	TCAAGAACTGGAAACGCAGC	GCCGGTAATGTGTAATGCGT
W303 region 2	AAGTTGTTTGACAGACCGGC	ACTTGTCGTCAGAGGAAGCA
Lactose transporter	AAACGTCGGCCAATTGTTCC	AGGGTGCAACAAATGTCCCC
Amino acid transporter	GTGGCATGGAATAGCGAACG	ACTTTGTGCGCCTTTTGACA
Proline activator	TGCCCGATGGTAGACACATC	AGCCAGGTGATCGCATCATT
IRT1	CACATGGATTCTCGGACTTTC	CTTGAGAAGCTCCAAGTACTGC
MEL1	GCTTTCTACTTTCTCACCGC	TCAAGAAGAGGGTCTCAACC
BIO6	TGCGTTCAGCAAGGATACAA	CCAAACAGCATACCCGTTCT
MPR1	CATTCATTCGCTGTCGTTGT	ATGAGGCACCAGTCCAATTC
TAT3	CCTGGTCCCTTTGCAAATTA	TGGAATACCCCTTTTGCTTG
RTM1	GGGATGCAAGAAAGGCTTC	GTACGGCGCTATCTCCTTTG
ARN2	TATTGCTATGTGGGCGTTCA	ACAAAGGGCCCGATAATCTT
Ty3	GTTACCACCAGATCCCGATG	CTGTTTTAGGCGTCGGAAAG
ARR3	GGAATCAGATTGCTGGAGGA	AAAGCACCAATGGGACAAAG
S. paradoxus		
BIO6	ACGGATTTGTCGCTGAAAAC	GTCTGGCACTATCCCTGCAT
MPR1	TCACTCATTCGCCGTTATTG	AGCATCCACGGGCTCACTAA
TAT3	GTCGTGGGCTGTAACCAAAT	CGTGGAAAATTGCATGTCTG
IRT1	CACATGGATTCTTGGCCTTT	AGAAAAAGCGAAACCAAGCA
ARN2	AGTCGGTAACATCCCACGAG	AGGAACATAGGCGCTGAGAA
Ty3	TGTAGCGGTCTTTTGGTTCC	TAATGATCTCCCGCCAAGAC
ARR3	CTAGATGCATTGCCATGGTG	TAATGCGAAGGTCGAAAACC
Amino acid transporter	GTCTACCATTAGCCAAGATAAGG	GTTACCTGCTGACCATGCCGATG
MEL1	GGATGGGACAATTGGAACAC	TCGTTCCAGCCACCTATACC
